# Astilbin Inhibits the Activity of Sortase A from *Streptococcus mutans*

**DOI:** 10.3390/molecules24030465

**Published:** 2019-01-28

**Authors:** Junxian Wang, Yan Shi, Shisong Jing, Haisi Dong, Dacheng Wang, Tiedong Wang

**Affiliations:** 1 School of Pharmaceutical Science, Jilin University, Changchun 130062, China; wangjx16@mails.jlu.edu.cn (J.W.); shiyan@jlu.edu.cn (Y.S.); 2 College of Animal Science, Jilin University, Changchun 130062, China; jingss18@mails.jlu.edu.cn (S.J.); adonghaisi@163.com (H.D.)

**Keywords:** *Streptococcus mutans*, astilbin, sortase A, biofilm, antivirulence

## Abstract

*Streptococcus mutans* (*S. mutans*) is the primary etiological agent of dental caries. The *S. mutans* enzyme sortase A (SrtA) is responsible for anchoring bacterial cell wall surface proteins involved in host cell attachment and biofilm formation. Thus, SrtA is an attractive target for inhibiting dental caries caused by *S. mutans*-associated acid fermentation. In this study, we observed that astilbin, a flavanone compound extracted from *Rhizoma Smilacis Glabrae*, has potent inhibitory activity against the *S. mutans* SrtA, with an IC_50_ of 7.5 μg/mL. In addition, astilbin was proven to reduce the formation of biofilm while without affecting the growth of *S. mutans*. The results of a molecular dynamics simulation and a mutation analysis revealed that the Arg213, Leu111, and Leu116 of SrtA are important for the interaction between SrtA and astilbin. The results of this study demonstrate the potential of using astilbin as a nonbactericidal agent to modulate pathogenicity of *S. mutans* by inhibiting the activity of SrtA.

## 1. Introduction

Dental caries is a common chronic disease that causes considerable anxiety, pain, tooth loss, malnutrition, and even disability across all age groups [1]. The disease process mainly involves bacteria, including *Streptococcus mutans*, *Streptococcus sanguis*, *Streptococcus sobrinus*, *Lactobacillus* spp., and *Streptococcus oralis* [2,3]. Among these bacteria, the Gram-positive bacterium *S. mutans* is the primary etiological agent of human dental caries [4], while also infecting cardiac endothelial cells and causing infective endocarditis [5]. *S. mutans* uses two adhesion mechanisms—sucrose-independent adhesion and sucrose-dependent adhesion. In the presence of sucrose, cell-wall-associated glucosyltransferases convert extracellular sucrose into glucan, which, together with glucan-binding proteins (Gbps) on the bacterial surface, facilitate cell–cell aggregation and the subsequent formation of dental biofilms on the tooth surface [6]. In the absence of sucrose, the adhesion of *S. mutans* to the dental surface, or other bacteria in dental plaque, is mediated by several surface adhesins. One of the primary adhesins of *S. mutans* is streptococcal protein antigen P (SpaP, also known as antigen I/II or P1), which can bind to salivary agglutinin glycoprotein (SAG) [7]. *S. mutans* lacking SpaP exhibited diminished adhesion to SAG-coated surfaces or to salivary pellicles in vitro, and monkey or human subjects immunized with antigen I/II exhibit reduced colonization by *S. mutans* [8]. Subsequent studies showed that SpaP and another adhesin, wall-associated protein A (WapA), can mediate *S. mutans* binding to collagen [9], suggesting that they have a role in the bacterial attachment to oral and other tissues. In addition to SpaP and WapA, glucan-binding proteins A (GbpA) and C (GbpC) also play crucial roles in biofilm formation on the tooth surface [10,11].

The *S. mutans* surface adhesins are anchored to the bacterial cell surface by the highly conserved transpeptidase, sortase A (SrtA) [12]. SrtA recognizes the sorting signal of surface proteins containing a highly conserved LPXTG motif (where X represents any amino acid) at the carboxy-terminal end of the protein and cleaves peptide bonds after the threonine. The released carboxy-terminus of threonine is attached to the pentaglycine of lipid II-surface protein. Lastly, surface protein-lipid II complex is affixed to the cell wall peptidoglycan via transglycosylation and transpeptidation reactions [13]. Moreover, the SrtA-deficient *S. mutans* strain cannot anchor the protein to the bacterial cell surface, and exhibits lower adherence to oral mucosa or teeth and decreased biofilm biomass on the tooth surface, reducing the formation of caries [14]. Thus, SrtA has an important role in the formation of dental caries by regulating the sorting of the adhesion-related protein to the cell surface, and is a promising target for drug development to prevent or treat dental caries. Inhibition of bacterial adherence is an ideal strategy to combat biofilm-related infections, because it can prevent biofilm establishment without changing the ecological balance within the oral cavity.

To date, many SrtA inhibitors have been identified, including synthetic small molecules [15,16], rationally designed peptide-analogs [17,18], and natural products derived from plants [19,20,21,22]. Among them, many flavonoids extracted from medicinal plants display good inhibitory activity against SrtA, including quercetin, which inhibits the *S. aureus* SrtA [19], epigallocatechin gallate, which inhibits the *S. pneumoniae* SrtA [20], and formononetin, which was found to be a potent inhibitor of *S. mutans* SrtA [21]. Huang et al. reported that morin, a flavonoid constituent of numerous Chinese herbs, can restrain the SrtA of *S. mutans*, thereby inhibiting the adhesion of *S. mutans* and reducing the consequent formation of biofilm [22].

**Figure 1:**
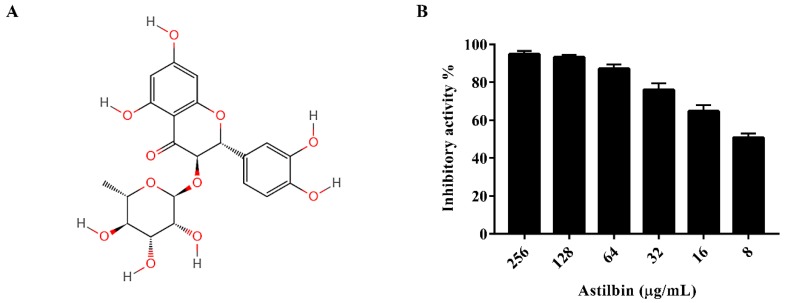
The structure of astilbin and the inhibition of *S. mutans* SrtA by astilbin in vitro. (**A**) The chemical structure of astilbin. (**B**) The inhibitory effect of astilbin against the SrtA of *S. mutans*. The purified SrtA was incubated with or without different concentrations of astilbin at 37 °C for 1 h, then the fluorescent substrate peptide was added, and the plate was incubated for 1 h. The fluorescence intensity of each well was measured with an excitation wavelength of 350 nm and an emission wavelength of 495 nm. The values indicate the mean values of three independent experiments. The error bars represent the standard deviations.

Astilbin is a naturally derived flavonoid compound isolated from *Rhizoma Smilacis Glabrae* (Figure 1A), which has been commonly used in traditional Chinese medical treatment. Astilbin has many properties, such as anti-*S. sobrinus* [23], anti-inflammatory [24], antioxidant [25], and immunosuppressive activities [26]. However, there are few reports on the inhibitory effects of astilbin on bacterial biofilms. In this study, we observed that astilbin can repress the activity of SrtA and the biofilm formation of *S. mutans*, indicating its potential for use as an oral biofilm inhibitor.

## 2. Results

### 2.1. Inhibition of S. mutans SrtA by Astilbin

The activity of SrtA was analyzed using a fluorescence resonance energy transfer (FRET) assay, as described in a previous study [27], with Abz-LPATG-Dap(Dnp)-NH_2_ used as the substrate peptide. The purified SrtA of *S. mutans* was incubated with the substrate peptide in the presence of various concentrations of astilbin in the reaction buffer. The results indicated that astilbin inhibited the activity of SrtA in a dose-dependent manner (Figure 1B), with an IC_50_ value of 7.5 μg/mL.

### 2.2. Antibacterial Activity of Astilbin

**Figure 2:**
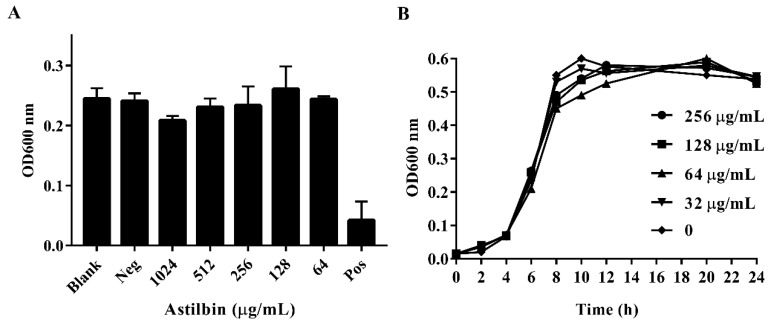
The minimum inhibitory concentration (MIC) of astilbin against *S. mutans* and the growth curves of *S. mutans* treated with astilbin. (**A**) The growth state of *S. mutans* in the presence of different concentrations of astilbin. “Neg” represents the negative control group and “Pos” represents the positive control group. The blank group contained only the brain heart infusion (BHI) broth and the tested *S. mutans*, the negative control group was treated with 1% dimethyl sulfoxide (DMSO), and the positive control group was treated with 20 μg/mL of chlorhexidine. (**B**) The growth curves of *S. mutans* treated with different concentrations of astilbin. The initial inoculum was approximately 1 × 10^6^ colony-forming units/mL. The growth rates were measured by determining the optical density (OD) every two hours for one day.

To determine if astilbin inhibits the growth of *S. mutans*, the minimum inhibitory concentration (MIC) of astilbin against *S. mutans* was determined, and *S. mutans* growth curves in the presence of astilbin were generated. As shown in Figure 2A, the MIC of astilbin against *S. mutans* was above 1024 μg/mL. Furthermore, the OD600 value of negative control (1% dimethyl sulfoxide (DMSO)) was similar to that of the blank control group, reflecting that there was no antimicrobial activity of the negative control. The growth curves showed that the growth of *S. mutans* treated with various concentrations of astilbin was similar to that of the untreated group (Figure 2B). These results suggest that astilbin does not affect the proliferation of *S. mutans* and will not lead to the development of bacterial drug resistance.

### 2.3. Inhibition of Biofilm Formation by Astilbin

**Figure 3:**
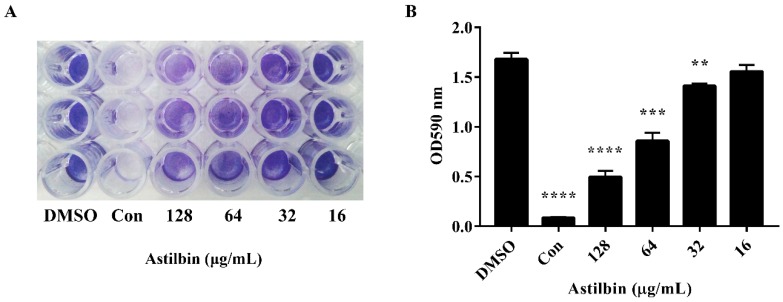
Inhibition of *S. mutans* biofilm formation by astilbin. (**A**) Photograph of *S. mutans* biofilms grown in the wells of a 96-well plate. Cells were cultured in BHI media containing different concentrations of astilbin for 16 h and stained with 0.1% crystal violet. The group treated with 128 μg/mL of morin served as a positive control (Con). The DMSO treatment group served as a negative control. (**B**) Quantification of the biomass of *S. mutans* treated with astilbin. The data were obtained from three independent experiments. Significant differences between groups were accepted at ** *p* < 0.01, *** *p* < 0.001, and ****p < 0.0001.

The attachment of *S. mutans* to tooth surfaces is the first step in biofilm formation [28], and SrtA inhibition should reduce the levels of cell surface proteins related to biofilm formation. Therefore, we further assessed the effect of astilbin on the *S. mutans* biofilm formation. The *S. mutans* biofilm biomass was measured after 18 h of growth on saliva-coated plates in a brain heart infusion (BHI) medium. As shown in Figure 3A, the experimental groups treated with 64 or 128 μg/mL of astilbin had a notable difference in biofilm integrity and thickness compared to the groups without astilbin. However, the biofilm treated with 32 and 16 μg/mL of astilbin did not show a difference from the controls. The quantitative results of biofilm formation were in line with the results of the crystal violet staining (Figure 3B). The groups treated with 64 μg/mL of astilbin showed a 49% reduction in biofilm formation, and the suppression effects of the 128 μg/mL groups (approximately 70%) were more obvious compared to the groups treated with 1% DMSO. The ability of *S. mutans* to form biofilms on plate surfaces was inhibited by astilbin in a dose-dependent manner.

### 2.4. Determination of the Molecular Mechanism

**Figure 4:**
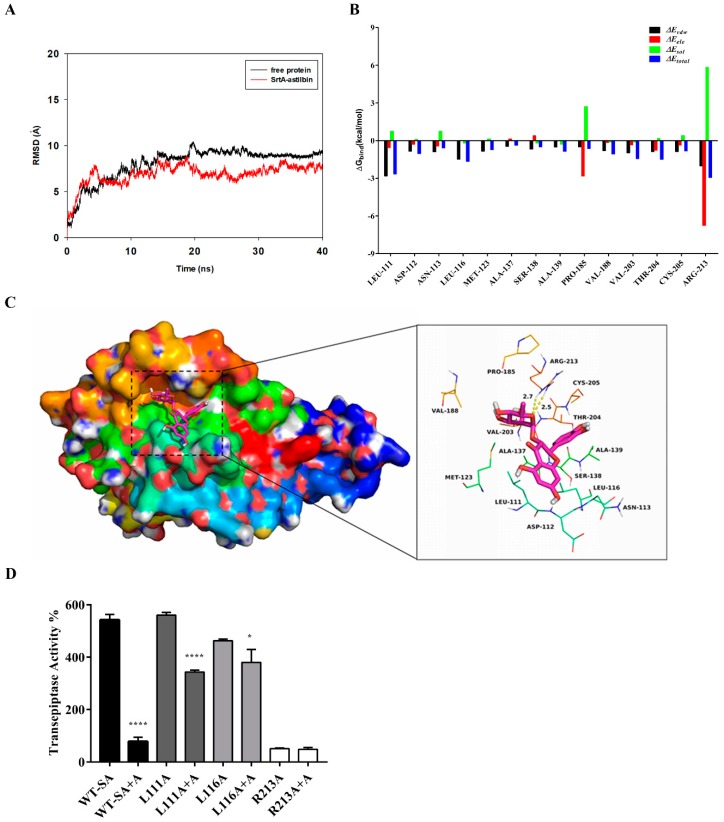
The results of the molecular docking (MD) simulation of the SrtA-astilbin complex. (**A**) The root-mean-square deviations (RMSD) exposed by the backbone atoms of the protein during the MD simulation of the SrtA-astilbin complex. (**B**) Decomposition of the binding free energy on a per residue basis in the SrtA-astilbin complex. (**C**) The predicted interaction mode of astilbin with the amino acid residues of the catalytic center of SrtA. (**D**) The inhibition effect of astilbin (64 μg/mL) (A represents astilbin) on the activities of SrtA and its mutants L111A-SrtA, L116A-SrtA, and R213A-SrtA. Significant differences between groups were accepted at * *p* < 0.05 and **** *p* < 0.0001.

Based on the above results, we performed a molecular docking (MD) simulation of the SrtA-astilbin complex to investigate the underlying molecular mechanism that inhibits the SrtA activity by astilbin. The potential binding mode of astilbin to the active site of SrtA was assessed via a 40 ns MD simulation using Autodock vina 1.1.2 and Amber 14. As shown in Figure 4A, the results of the root-mean-square deviation (RMSD) assay showed that the system became an equilibrium state after 40 ns of the simulation. To obtain more information regarding the contributions of the residues surrounding the binding site to the system, the binding free energies (Δ*G_bind_* in kcal/moL) between these residues and astilbin were calculated. As shown in Figure 4B, in the SrtA-astilbin complex, the Arg213 residue has a strong electrostatic (∆*Eele*) interaction with astilbin, with an ∆*Eele* value of < −6.5 kcal/moL. Further analysis revealed that the Arg213 residue is oriented toward the rhamnose group of astilbin, leading to the formation of two strong hydrogen bonds (bond lengths of 2.5 and 2.7 Å) between SrtA and astilbin (Figure 4C). In addition, the residues Arg213, Leu111, and Leu116, with Van der Waals (∆*E_vdw_)* values of < −1.5 kcal/moL, have notable Van der Waals contributions due to their proximity to the astilbin molecule, indicating that these three residues are crucial for the binding of astilbin to SrtA. In addition, the total binding energy of the SrtA-astilbin complex was calculated, and a ∆*G_bind_* value of –28.8 kcal/moL was determined for astilbin, suggesting that it can strongly bind to and interact with SrtA.

To further validate the results of the MD simulation, three mutants were constructed—L111A-SrtA, L116A-SrtA, and R213A-SrtA. The mutant proteins were subsequently expressed and purified, and the inhibitory activity of astilbin on these mutated proteins was assessed via a FRET assay. As shown in Figure 4D, the mutation of Arg213 resulted in a significant decrease in the transpeptidase activity, indicating that it is a crucial amino acid residue in SrtA. Moreover, the inhibitory activity of astilbin against the mutated proteins decreased sharply compared with the wild-type SrtA (WT-SA). These results were in agreement with those obtained by the MD simulation, validating the reliability of these results.

## 3. Discussion

Many studies have shown that isogenic SrtA knockout strains exhibit a notable reduction in the ability to anchor surface proteins containing the canonical LPXTG motif, and are less lethal than wild-type strains in various animal models of infection [29,30]. Additionally, the SrtA-mutants of *S. mutans* show a decreased ability to attach to human extracellular matrix proteins and to colonize the murine oral cavity and teeth [31]. As the primary aetiological agent of dental caries, *S. mutans* forms biofilm aggregates on the surfaces of teeth with the assistance of many cell surface-localized and secretory factors. Early studies demonstrated that a few compounds from Chinese traditional medicine, such as morin [22], curcumin [32], trans-chalcone [33], and metabolites from the flowers of *Sophora japonica* [34], can inhibit *S. mutans* biofilm formation by inhibiting SrtA activity. In this study, when SrtA of *S. mutans* was incubated with different concentrations of astilbin, the SrtA catalytic activity decreased in a concentration-dependent manner (Figure 1B). In addition, even increasing the concentration of astilbin to 1024 μg/mL, did not decrease the bacterial growth rate (Figure 2A). Thus, astilbin exerts little selective pressure on *S. mutans*, and is unlikely to induce the development of resistance. To further evaluate the inhibitory effects of astilbin on the biofilm formation of *S. mutans*, we used the crystal violet staining method to quantify biofilm biomass. The quantitative results (Figure 3) demonstrated that 64 μg/mL of astilbin could inhibit the biofilm formation of *S. mutans* in a concentration-dependent manner. There was a 50% and a 70% reduction of the biofilm biomass after 16 h in the presence of 64 and 128 μg/mL of astilbin, respectively. The in vitro IC_50_ value and biofilm inhibition concentrations are inconsistent, because SrtA activity is not completely inhibited at this concentration, so a small amount of surface protein is still present on the bacterial surface, indicating that a higher drug concentration may be required to completely inhibit biofilm formation.

The results of the MD simulation and the SrtA mutation analyses indicated that astilbin binds to SrtA via electrostatic, hydrogen bond, and Van der Waals interactions, and the amino acid residues Arg213, Leu111, and Leu116 play important roles in the interaction between SrtA and astilbin (Figure 4B–D). The relative positions of key active-site residues (His, Cys, Arg) are highly conserved in sortase enzymes from Gram-positive bacteria. Wallock-Richards et al. elucidated that, similar to other SrtA enzymes, the crystal structure of the SrtA of *S. mutans* showed that Cys205, Ala139, and Arg213 are located in three adjacent β-strands, which form a tunnel-like hydrophobic pocket [33]. The strong interaction of astilbin with Arg213 interferes with the catalytic activity of SrtA, and results in reduced anchoring of cell surface proteins.

The in vitro study here and other studies showed that the inhibition of SrtA can weaken the biofilm formation of *S. mutans*. However, as the pathogenesis of caries involves other bacterial species, the practical effects of in vivo use of SrtA inhibitors still needs further evaluation.

In summary, our results demonstrated that astilbin can inhibit the activity of SrtA by interacting with crucial amino acid residues and disrupt biofilm formation of *S. mutans.* Thus, astilbin is a promising lead compound to develop products that can be incorporated into oral care products, such as mouth rinses or toothpastes, to enhance their anticaries properties.

## 4. Materials and Methods

### 4.1. Bacteria, Chemicals, and Growth Conditions

The *S. mutans* strain (ATCC 25175) used throughout this study was purchased from the American Type Culture Collection (ATCC, Manassas, VA, USA). Bacteria were anaerobically cultured in a BHI broth (Sigma) at 37 °C with shaking. Astilbin was purchased from the Chengdu Herbpurify Corporation (Chengdu, China) with a purity > 98%. Astilbin was dissolved in dimethyl sulfoxide (DMSO) (Sigma-Aldrich, St. Louis, MO, USA) to 102.4 mg/mL as a colorless, transparent solution, and stored at 4 °C until needed. The substrate peptide Abz-LPATG-Dap(Dnp)-NH_2_ (Abz: ortho-aminobenzoic acid; Dnp: 2,4-dinitrophenyl) was synthesized by GL Biochem (Shanghai, China).

### 4.2. Cloning and Expression of the SrtA and Its Mutants

A 741 bp DNA fragment encoding the SrtA gene was amplified by polymerase chain reaction (PCR) from the *S. mutans* chromosome using the primer pairs SmsrtA-F/SmsrtA-R, the amplified fragment was digested with BamHI and XhoI, and cloned into the vector pET28a, yielding pET28SmsrtA. Point mutations in the gene-encoding SrtA were generated using a QuikChange site-directed mutagenesis kit (Stratagene, San Diego, CA, USA) to yield L111A-SrtA, L116A-SrtA, and R213A-SrtA. The genes of point mutations were confirmed by sequencing by Sangon Biotech Co., Ltd (Shanghai, China). To obtain recombinant wild-type and mutant SrtA proteins, *Escherichia coli* strain BL21 (DE3) was transformed with pET28SmsrtA and the SrtA mutant constructs. Protein expression was induced with 0.5 mM isopropylthio-β-d-galactoside (IPTG) (Sigma) at the mid-log-phase for 12 h at 16 °C, respectively. The soluble His-tagged wild-type and mutant SrtA proteins were further purified using the Ni-NTA system, as described in a previous study [13]. All primers used in this study are listed in Table 1.

### 4.3. Determination of MIC

*S. mutans* was grown overnight on BHI agar plates under anaerobic conditions. The MIC values of astilbin against *S. mutans* were measured using the two-fold serial dilution method, following the Clinical Laboratory Standards Institute (CLSI) guidelines [35]. The astilbin stock solution was serially diluted with a BHI broth from 1024 to 64 μg/mL in a 96-well flat-bottomed plate under aseptic conditions. The BHI broth with 20 μg/mL of chlorhexidine was used as a positive control, and 1% DMSO served as a negative control. After adding 1 μL of the prepared bacterial suspension (1 × 10^7^ colony-forming units (CFU)/mL) equally into each well, which contained 200 μL of the BHI broth and serial-diluted astilbin, the plate was placed in the anaerobic incubator at 37 °C for 16 h. Absorbance values at 600 nm were determined using a microplate reader (Infinite^®^ F500, Tecan, Shanghai, China). The MIC was defined as the minimal concentration at which a visible growth of microorganisms was inhibited under defined growth conditions [36]. All experiments were repeated three times.

### 4.4. Growth Curves of S. mutans

*S. mutans* cultures were grown aerobically overnight at 37 °C in a BHI medium. After culturing, 100 μL of the bacterial cultures (1 × 10^7^ CFU/mL) were transferred to aseptic test flasks containing 10 mL of a sterilized BHI medium and different concentrations of astilbin (0, 32, 64, 128, 256 μg/mL). All the flasks were aerobically cultured at 37 °C. The optical density of *S. mutans* was recorded at 600 nm every two hours for one day, with samples being vortexed before measuring.

### 4.5. SrtA Activity Assay

The SrtA activity inhibition assay was performed according to a previously published protocol, with slight modifications [13]. Briefly, the inhibition of SrtA activity of *S. mutans* by astilbin was determined by quantifying the fluorescence intensity, which changes with the cleavage of the fluorescent substrate peptide Abz-LPATG-Dap(Dnp)-NH_2_. The assay was conducted in a 200 μL reaction mixture containing buffer A (50 mM Tris-HCl, 300 mM NaCl, 5 mM CaCl_2_, pH 8.0), 7 μM of SrtA, and various concentrations of astilbin (256, 128, 64, 32, 16, 8 μg/mL). The reaction mixture was incubated for 1 h at 37 °C, after which 10 μM of Abz-LPATG-Dap(Dnp)-NH_2_ was added, and the reaction was incubated for another hour at the same temperature. To exclude the influence of the fluorescence of astilbin, the same reaction mixture without SrtA was used as a negative control and treated exactly in the same way. In addition, the blank control group (containing buffer A, 10 μM of substrate peptide, and 7 μM of SrtA) without astilbin was used to indicate the initial activity of SrtA. Each astilbin concentration, or well, had three parallel repetitions. The fluorescence values were measured and the percentage inhibition of SrtA by astilbin was calculated using the following equation: % inhibition = *F_blank_*– (*F_sample_* – *F_negative_*)/*F_blank_* × 100%, where *F_negative_* is the fluorescence value of the negative control group, *F_sample_* is the fluorescence value of the astilbin-treated group, and *F_blank_* is the fluorescence value of the blank control [37]. The experiments were repeated at least three times independently.

### 4.6. Crystal Violet Biofilm Assay

Nonstimulated saliva from healthy rats was collected in sterile 50 mL centrifuge tubes, and clarified by centrifugation (16,000× *g*, 4 °C, 10 min). The supernatant after centrifugation was filtered with a 0.22 μm Millipore filter. The 96-well microtiter plates (Corning Costar Co., Cambridge, MA, USA) were coated with 200 μL of saliva-buffer mixture for 1 h at 37 °C, according to the procedure of Islam et al. [38] with minor modifications. Subsequently, the liquid was discarded aseptically, and 1 μL of an overnight culture of *S. mutans* (1 × 10^7^ CFU/mL) was inoculated into 200 μL of sterile BHI broth supplemented with different concentrations of astilbin (128, 64, 32, 16 μg/mL). *S. mutans* cultured with the BHI broth with 128 μg/mL of morin served as a positive control. For each astilbin concentration, three parallel wells were assayed. After static incubation for 16 h at 37 °C anaerobically, the media and unattached cells were discarded gently, and the wells were washed three times with PBS. Then the biofilms were stained with 100 μL of 0.1% crystal violet for 15 min at 37 °C, after which the unbound component was rinsed twice with double distilled water. Subsequently, the crystal violet stain was dissolved in 200 μL of 33% acetic acid. The absorbance at 570 nm was determined using a microplate reader (Infinite^®^ F500, Tecan, China) to quantify the biofilm biomass.

### 4.7. Molecular Docking and Molecular Dynamics Simulation

Autodock Vina 1.1.2 [39] was used to elucidate the binding mode between astilbin and *S. mutans* SrtA. The SrtA structure (PDB ID:4TQX) was obtained from the Research Collaboratory for Structural Bioinformatics (RCSB) Protein Data Bank. The astilbin structure was generated using ChemBio3D Ultra 12.0 (ChemBioOffice, New Jersey, CA, USA). For Vina docking, the parameters were maintained at the default settings, unless otherwise mentioned. After docking, the most stable pose of the SrtA-astilbin complex that was obtained was used in an MD simulation analysis using Amber 14 [40,41,42]. The astilbin structure was first prepared using ACPYPE [43] to generate automatic topologies. The forcefield “leaprc.gaff” was selected for astilbin, while “leaprc.ff14SB” was selected to prepare SrtA. Before starting the simulation, equilibration of the solvated SrtA-astilbin complex was performed using a short minimization. Finally, the 40 ns MD simulation was performed on a Dell Precision T5500 workstation. Furthermore, the ligand-protein binding free energies (*ΔG_binding_* in kcal/moL) were calculated using the analyze tool in AmberTools 15 [44]. For each complex, the binding free energy was calculated using the following equation: Δ*G_binding_* = *G_complex_*‒ *G_protein_* ‒ *G_ligand_*, where *G_complex_*, *G_protein_,* and *G_ligand_* are the free energies of the SrtA-astilbin complex, the SrtA protein, and the ligand astilbin, respectively.

### 4.8. Statistical Analysis

All experiments were performed in triplicate. The statistical analysis of the data was performed using a one-way ANOVA and *t*-tests with GraphPad Prism7 software (GraphPad software Inc, La Jolla, CA, USA). All data of the three independent experiments were shown as mean ± SD. Statistical significance was determined when *p* < 0.05.

## Figures and Tables

**Table 1:** Primers used in this study.

Primer Name	Sequences (5′–3′)
SmsrtA-F	CGCGGATCCATGAAAAAAGAACGTCAATCTAGGA
SmsrtA-R	CCGCTCGAGTTAAAATGATATTTGATTATAGGACTGCC
R213A-SrtA-F	TTGTTCATGGCACATATAAGGGGGAA
R213A-SrtA-R	TTGTTGCAGCAGTCGCCC
L111A-SrtA-F	AAGGAGCAGATAATGTTGGCTTAAC
L111A-SrtA-R	TGAAGATTGGTAAATTGATTTTTAAGTCTGG
L116A-SrtA-F	TTGGCGCAACATATGGTG
L116A-SrtA-R	ATTTATCTAATCCTTTGAAGATTGGTAAATTG

The underlined basic groups represent restriction endonuclease recognition sites or mutated codons.
